# Spectrum and Prevalence of Thyroid Diseases at a Tertiary Referral Hospital in Mogadishu, Somalia: A Retrospective Study of 976 Cases

**DOI:** 10.1155/2021/7154250

**Published:** 2021-12-26

**Authors:** Mohamed A. Hassan-Kadle, Abdulkamil Abdullahi Adani, Hasan Huseyin Eker, Esra Keles, Marian Muse Osman, Hussein Mahdi Ahmed, Şeyma Görçin Karaketir

**Affiliations:** ^1^SomGastro Clinic, Center for Digestive and Liver Disease, College of Medicine & Health Science, Abrar University, Mogadishu, Somalia; ^2^Department of Internal Medicine, University of Health Sciences Turkey, Mogadishu Somalia-Turkey Recep Tayyip Erdoğan Training and Research Hospital, Mogadishu, Somalia; ^3^Department of Public Health, University of Health Sciences Turkey, Hamidiye Faculty of Medicine, Istanbul 34668, Turkey; ^4^Department of Gynecologic Oncology, University of Health Sciences Turkey, Zeynep Kamil Training and Research Hospital, Istanbul 34668, Turkey; ^5^Department of Public Health, University of Health Sciences Turkey, Mogadishu Somalia-Turkey Recep Tayyip Erdoğan Training and Research Hospital, Mogadishu, Somalia; ^6^Occupational Health Training Programme, Department of Public Health, İstanbul University, İstanbul School of Medicine, İstanbul, Turkey

## Abstract

**Background:**

Thyroid disorder is one of the most common noncommunicable diseases worldwide and neglected public health issues in Somalia. The aim of the study thus was to investigate the thyroid disorders in patients attending to the largest tertiary referral hospital in Somalia.

**Methods:**

This retrospective study was conducted on patients admitted to the internal department of Somalia Mogadishu-Turkey Education and Research Hospital, Somali, between January 2017 and December 2019. Patients who were tested for thyroid function tests and had complete data were included. Patients with incomplete data and currently treated for any thyroid disorder were excluded from the study. Abstracted data including patients' sociodemographic characteristics, thyroid function tests, and histopathological findings were retrieved from the hospital database system.

**Results:**

A total of 976 patients with thyroid disorders were enrolled, of whom 66.6% (*n* = 650) were female and 33.4% (*n* = 326) were male. The mean age of the patients was 47 ± 18.5 years. The majority of the patients were reported in the 31–50 (35.9%) age range. The most reported thyroid function disorders were 58.8% euthyroid sick syndrome followed by 15.4% hypothyroidism, 12.5% subclinical hypothyroidism, 7.6% hyperthyroidism, and 5.7% subclinical hyperthyroidism. The distribution of comorbidity indicated that 13.4% had diabetes mellitus, 10.4% had HIV, 4.9% had malaria, and 4.5% had HIV and malaria coinfection. Thyroid malignancies were detached in 22 (2.2%) patients including eleven papillary thyroid cancer, nine patients had follicular thyroid cancer, and two patients had differentiated thyroid cancer.

**Conclusions:**

Euthyroid sick syndrome was the most common type of thyroid disease in our setup. Hypothyroidism is the second most common, followed by subclinical hypothyroidism. Papillary thyroid cancer was the predominant histology among thyroid malignancies, followed by follicular thyroid cancer. This study revealed that thyroid diseases emerge as an important endocrine disorder encountered in Somali, necessitating a major public health response.

## 1. Introduction

Thyroid diseases are one of the most important public health problems worldwide ranking second in endocrine diseases after diabetes mellitus including Africa [1, 2]. In Africa, they were rare particularly in the early 1960s, although the 1970s saw an increase in reported cases of thyroid diseases in Africans, and iodine deficiency was also noted to be the most common cause of thyroid disorders [3, 4].

Iodine is a trace element in the synthesis of thyroid hormones, and the thyroid gland secretes two metabolic hormones, thyroxine (T4) and triiodothyronine (T3), regulating metabolic rate, growth, and development [5, 6]. These hormones produced by the thyroid gland are imbalanced in the regulation which can cause many disorders including hypothyroidism, subclinical hypothyroidism, hyperthyroidism, subclinical hyperthyroidism, and secondary hypothyroidism [6, 7].

Globally, the main micronutrients including iodine are a public health problem affecting all segments of the population. Sub-Saharan Africa had been classified as an area of moderate-to-severe iodine deficiency for a long time. Therefore, the thyroid disease burden in Africa represents above 25% of the worldwide [4]. Somalia is a coastal country that has the second longest coastline in Africa. It is also one of the African countries where groundwater is the primary source of dietary iodine. But the deficiency of micronutrients including iodine among the Somalis population is due to the limited access to iodized salt and high existence of cultural beliefs and barriers that delay the consumption or inadequate intake of these micronutrients including iodine [8–10].

This study was to document all types of thyroid diseases seen in the largest referral hospital in the country to be the baseline data for future research studies about this disease; to the best of our knowledge, this is the first study to be reported from Somalia before and after the Civil War.

## 2. Materials and Methods

We retrospectively analyzed the records of patients with thyroid disorder who were admitted to the internal medicine department of Somalia Mogadishu-Turkey Recep Tayyip Erdogan Training and Research Hospital, Somalia, between January 1, 2017, and December 31, 2019. Our hospital is the largest multidisciplinary tertiary referral healthcare center of the region. This study was approved by the research ethics committee of Somali Mogadishu-Turkey Recep Tayyip Erdogan Training and Research Hospital (13.05.2020-MSTH/3790). The database management was in accordance with privacy legislation, and the presented study was in accordance with the ethical principle of the Declaration of Helsinki.

Data were obtained through hospital's electronic database system for the records of patients diagnosed with thyroid disease in our hospital. Patients diagnosed with thyroid disease and with complete data were included in the study. Patients with incomplete data and currently treated for any thyroid disorder were excluded from the study.

Abstracted data included patients' demographic and clinical characteristics (age, gender, and comorbidities), histopathological findings, and the results of thyroid function tests consisting of thyroid-stimulating hormone (TSH), free thyroxine (FT4), and free triiodothyronine (FT3). Histopathological findings were classified as benign and malignant. Papillary thyroid cancer, follicular thyroid cancer, and differentiated thyroid cancer were defined as malignant pathology.

The patients were categorized into 5 groups as subclinical hypothyroidism, hypothyroidism, euthyroid sick syndrome, subclinical hyperthyroidism, and hyperthyroidism, and their definitions were defined according to the American Association of Clinical Endocrinologists (AACE) in association with American Thyroid Association (ATA) guidelines 2013 [11]. The normal range for thyroid function assays in our institution is TSH 0.35–5.10 *μ*IU/mL, FT4 0.60–1.20 ng/mL, and FT3 1.80–4.20 pg/mL. Hypothyroidism was defined to FT4 < 0.60 ng/mL and TSH > 5.1 *μ*IU/mL, and subclinical hypothyroidism was defined as normal free hormone levels and a TSH > 5.1 *μ*IU/mL. Hyperthyroidism was defined as FT4 > 1.20 ng/mL and TSH < 0.35 *μ*IU/mL, and subclinical hyperthyroidism was defined as normal free hormone levels and TSH < 0.35 *μ*IU/mL. Euthyroid sick syndrome was defined as FT3 < 1.80 pg/mL, TSH < 5.10 *μ*IU/mL, and FT4 < 0.60 ng/mL. Thyroid function tests were measured by a Roche e411 Immunoassay Analyzer (Roche Diagnostics Corporation, Indianapolis, IN).

All statistical analyses were performed using SPSS software version 23. Descriptive analyses were presented using means, standard deviations, and median values. The proportions were presented using tables of frequencies and percentages. The chi-square test or Fisher's exact test, where appropriate, was used to compare these proportions in different groups using the Bonferroni correction to adjust for multiple comparisons. The variables were investigated using visual (histograms) and analytical methods (Kolmogorov–Smirnov) to determine whether or not they are normally distributed. Kruskal–Wallis and Mann–Whitney *U* tests were used to compare the parameters between the groups. A *p* value of less than 0.05 was considered statistically significant.

## 3. Results

**Figure 1:**
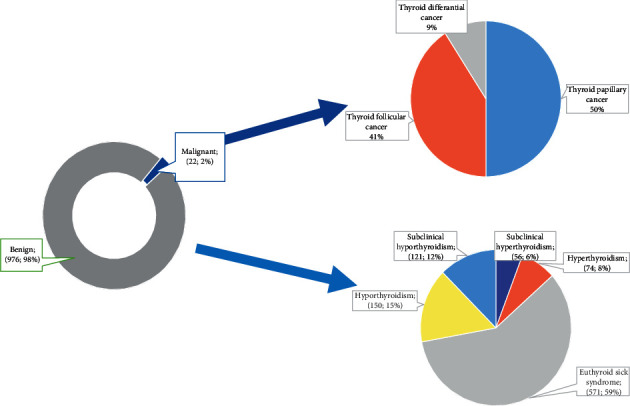
Distribution of thyroid disease cases (*n* = 976) by histopathological results and thyroid function disorders' subgroups, January 2017–December 2019, Somalia.

A total of 976 patients with thyroid disorders were enrolled in this study, of whom 66.6% (*n* = 650) were female and 33.4% (*n* = 326) were male. The mean age of the patients was 47 ± 18.5; most of the patients were in the age range of 31–50 (35.9%; *n* = 350). Distribution of the patients by thyroid function disorders showed that the majority of patients have euthyroid sick syndrome (58.8%; *n* = 571), while 15.4% (*n* = 150) of the patients were diagnosed with hypothyroidism, 12.5% (*n* = 121) were diagnosed with subclinical hypothyroidism, 7.6% (*n* = 74) were diagnosed with hyperthyroidism, and 5.7% (*n* = 55) were diagnosed with subclinical hyperthyroidism. When the distribution of comorbidity among 972 patients was examined, it was observed that 13.4% had diabetes mellitus (DM), 10.4% had HIV, 4.9% had malaria, and 4.5% had HIV and malaria coinfection. The characteristics of the study population are presented in Table 1. Thyroid malignancies were identified in 22 (2.2%) patients. Half of these patients had papillary thyroid cancer (*n* = 11). Nine patients had follicular thyroid cancer, and two patients had differentiated thyroid cancer. Additionally, the distribution of thyroid carcinoma subtypes can be seen in Figure 1.

There was a statistically significant difference between thyroid function disorders and gender (*p* ≤ 0.001). The euthyroid sick syndrome had been defined in 68.8% of males, while this was 53.8% in females. However, subclinical hyperthyroidism (6.3%), hyperthyroidism (9.6%), hypothyroidism (16.4%), and subclinical hypothyroidism (13.9%) were more common in females than in males. Age distribution in thyroid dysfunctions showed a statistically significant difference (*p* ≤ 0.001). The mean age of the hyperthyroidism group was 38 ± 14.6 years, the euthyroid sick syndrome group was 49 ± 19.6 years, and subclinical hypothyroidism was 48 ± 18.8 years. Of the 22 patients with thyroid cancer, 11 patients were in the euthyroid sick syndrome group, five patients were diagnosed with hypothyroidism, five patients were diagnosed with subclinical hypothyroidism, and one patient was diagnosed with subclinical hyperthyroidism. Euthyroid sick syndrome constitutes the majority of patients in all comorbidity groups, while 15% of patients with malaria were diagnosed with hypothyroidism, 15% subclinical hypothyroidism, 6% subclinical hyperthyroidism, and 1% hyperthyroidism; this rate was 8.5%, 21.5%, 6.4%, and 0% in HIV, respectively. Additionally, it was 11.5%, 12.3%, 11.5%, and 7% in DM, respectively. The distribution of the patients by thyroid function disorders is shown in Table 2.

The distribution of thyroid function disorders according to benign histopathology was as follows: euthyroid sick syndrome 59%, hypothyroidism 15%, subclinical hypothyroidism 12%, hyperthyroidism 8%, and subclinical hyperthyroidism 6%. The distribution of malignant thyroid cancer subtypes can be seen in Figure 1.

**Figure 2:**
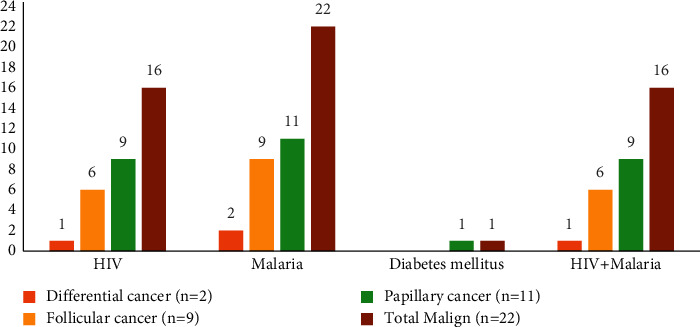
Distribution of thyroid cancers by comorbidities (*n* = 22), January 2017–December 2019, Somalia.

The distribution of thyroid cancers by comorbidities indicated that all patients had malaria, 16 patients had HIV, and one patient had DM. In addition, all patients with HIV also had a diagnosis of malaria. All 11 patients with papillary thyroid cancer had malaria, 9 had HIV, and one person had DM. When the distribution of follicular thyroid cancer (*n* = 9) by comorbidity was examined, it was seen that all of them had malaria, and 6 had malaria and HIV coinfection. Among the two patients diagnosed with differentiated thyroid cancer, one had malaria infection, and the other had both malaria and HIV coinfection. The distribution of thyroid cancers by comorbidities is presented in Figure 2.

**Figure 3:**
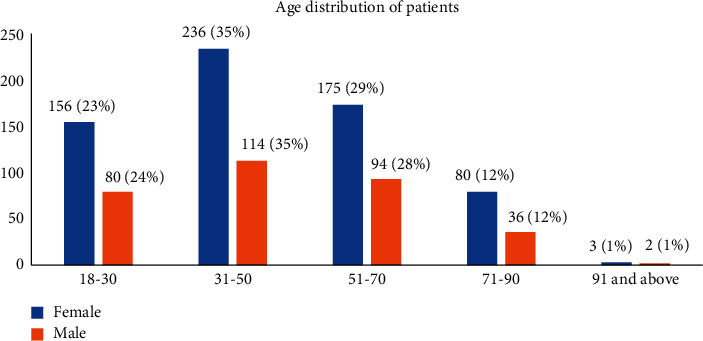
Age distribution of patients by gender (*n* = 976), January 2017–December 2019, Somalia.

The age distribution of thyroid patients by gender revealed that the most common age group in males and females was between 31 and 50, with a rate of 35%. The age group distribution showed a similar distribution between genders. The most common age groups were 51–70, 18–30, 71–90, and 91 and above, respectively. The age distribution of the patients by gender is presented in Figure 3.

## 4. Discussion

Thyroid disorder is a neglected major public health issue and the most common noncommunicable disease in developing countries [12]. Thyroid disease is one of the most common endocrine disorders worldwide ranking second in endocrine diseases after diabetes mellitus including Africa [1, 2]. This is the first study to examine the course of thyroid diseases in Somalia before and after the Civil War. This present study showed that the female population was common than the male population, and similar studies showed that the female population was predominant in Ethiopia, Kenya, Ghana, Nigeria, and Yemen and in sub-Saharan countries and the Middle East [4, 7, 13–17]. Thyroid disorder is the most common disease of the endocrine system which is increasing predominantly among females in the world [18]. Our study also showed that the mean age is 47 years, in line with studies in Ghana (43 years), Nigeria (42 years), Nepal (39 years), and Yemen (37 years) [4, 6, 19], and the most age group of our study was 31–50, while in Nepal, it was 31–45 years [6].

In the present study, we found that the most common type of thyroid disorder is euthyroid sick syndrome (58.8%). Euthyroid sick syndrome is characterized by the changes in the thyroid hormonal level in acute and chronic illness due to nonthyroid illness caused by the inhibition of an enzyme called 5-deiodinase by various pathogeneses which catalyzes T4 to T3 conversion. While low T3 levels and high T4 and rT3 levels were seen in acute changes, low T3, T4, and TSH levels were seen in chronic changes. Therefore, euthyroid sick syndrome is seen mostly in severe critical illness followed by major surgeries and inpatient or intensive care settings [20, 21]. A possible explanation for the majority of patients with euthyroid syndrome in our study is that our hospital is the largest multidisciplinary tertiary hospital in the region to which patients with chronic diseases were referred.

The second most common thyroid disease was primary hypothyroidism (15.4%), followed by subclinical hypothyroidism (12.5%), which was similar but more than the percentage found in a Nepal study that showed hypothyroidism was 29.6% and subclinical hypothyroidism was 28.3% [6]. Okpara et al. reported a higher frequency of primary hypothyroidism (4.9%) and subclinical hypothyroidism (6.3%) [15]. This high frequency of hypothyroidism may be explained due to poor iodine nutrition, cultural beliefs, and barriers that delay the consumption or inadequate intake of these micronutrients including iodine [8]. Our study also showed that hyperthyroidism and subclinical hyperthyroidism were 7.6% and 5.7%, respectively. Similarly, Okpara et al. reported a prevalence of 13.7% and 4.9% in hyperthyroidism and subclinical hyperthyroidism, and Agrawal et al. also reported 13.7% and 3.3 [6, 15]. Initiating programs such as the provision of enriching foods with iodine, prenatal Fe, and iodine vitamin supplements, raising public awareness of the disease either through mass media (TV, radio, and social media) or through posters in healthcare centers, and providing nutrition counseling and initiating plans of action to increase health literacy in the society are necessary for the prevention of thyroid disease in the early stages.

The current study showed the association between the gender and type of thyroid disease; the euthyroid sick syndrome was common in males (68.8%), which was similar to the study conducted by Agrawal et al. in Nepal that reported a prevalence of 73.9% [6]. However, subclinical hyperthyroidism (6.3%), hyperthyroidism (9.6%), hypothyroidism (16.4%), and subclinical hypothyroidism (13.9%) were more common in females than in males. Although the study in Turkey reported similar hypothyroidism and subclinical hypothyroidism in females than males, subclinical hyperthyroidism and hyperthyroidism were nearly equal in both of the genders [22].

Our study showed that the age distribution in thyroid diseases was a statistically significant difference (*p* ≤ 0.001). The oldest mean age of this study was seen in euthyroid sick syndrome according to the types of thyroid disease to reason that the euthyroid sick syndrome is a common disorder in the hospitalized older people and increases with increasing age [23]. The oldest mean age of this study was seen in subclinical hypothyroidism, while the youngest mean age was seen in hyperthyroidism groups.

Diabetes mellitus is the most prevalent disorder in endocrinological diseases, while thyroid disorders were the second disease. Therefore, there is a link between thyroid dysfunction and diabetes which was documented in several studies that they are close diseases. Diabetes mellitus is associated with a higher prevalence of thyroid disease, and vice versa [24]. Studies in Nepal among thyroid comorbid DM were shown in category three after the depression and hypertension [25], although our study observed that 13% of thyroid patients had diabetes mellitus which was the first category before HIV and malaria.

Thyroid malignancies are the most common endocrine tumors accounting for more than 90% of endocrine tumors. Papillary thyroid cancer is the most common type of thyroid malignancy, comprising 60–65% of all thyroid cancers [26]. Follicular thyroid cancer is the second most common malignancy accounting for 15 of the cases [27]. Our study showed that papillary thyroid cancer was the most common type followed by follicular thyroid cancer, and this was similar to the UAE [28], Saudi Arabia [29], and Yemen [30], while in Sudan, the most common type of thyroid cancer was follicular thyroid cancer [31], and in Pakistan, the most type seen was papillary thyroid cancer followed by medullary carcinoma, and follicular thyroid cancer is the third most common thyroid malignancy [32].

The limitations of this study are that, first, it is a single-centered study, while the second limitation of our study is the retrospective design with a small sample size. Larger population studies are needed in order to establish the epidemiology of thyroid disorders in our setup, but this study will be the baseline for future studies although this study is the first study of the country before and after the Civil War.

## 5. Conclusion

Thyroid disorders are one of the most common endocrine abnormalities encountered in our clinical practice. The present study demonstrated female predominance in thyroid disease, with the exception of the euthyroid sick syndrome in the male population, particularly in the elderly. The clinical profile of thyroid disease of this study comprises a wide range of disorders including hypothyroidism and subclinical hypothyroidism, hyperthyroidism and subclinical hyperthyroidism, euthyroid sick syndrome, and euthyroid. Euthyroid sick syndrome was the most common type of thyroid disease in our setup. Hypothyroidism was the second most common, followed by subclinical hypothyroidism. Papillary thyroid cancer was the predominant histology of thyroid malignancy followed by follicular thyroid cancer. Our research could be a valuable tool for decision makers in solving the burden of thyroid disease. Also, the present study sheds light on the clinical profile of thyroid disease by suggesting preventive public health strategies. Therefore, implementation of public health programs consisting of the provision of fortified food with iodine, prenatal Fe, and iodine vitamin supplements and raising public awareness of this disease through mass media may be useful in the prevention of thyroid disorders.

## Figures and Tables

**Table 1:** Baseline characteristics of the patients (*n* = 971), January 2017–December 2019, Somalia.

	*n* (%)

Gender	
Female	650 (66.6)
Male	326 (33.4)
Age (years, mean ± SD)	47 ± 18.5
Age groups	
18–30	236 (24.2)
31–50	350 (35.9)
51–70	269 (27.6)
71–90	116 (11.9)
≥91	5 (0.5)
Diagnosis	
Subclinical hyperthyroidism	55 (5.7)
Hyperthyroidism	74 (7.6)
Euthyroid sick syndrome	571 (58.8)
Hypothyroidism	150 (15.4)
Subclinical hypothyroidism	121 (12.5)
Comorbidities	
DM	131 (13.4)
HIV	101 (10.4)
Malaria	48 (4.9)
HIV and malaria	44 (4.5)
Type	
Benign	954 (97.8)
Malignant	22 (2.2)
Differentiated	2 (0.2)
Follicular	9 (0.9)
Papillary	11 (1.1)

**Table 2:** Baseline characteristics of the patients according to thyroid function disorders (*n* = 971), January 2017–December 2019, Somalia.

	Subclinical hyperthyroidism (*n* = 55)	Hyperthyroidism (*n* = 74)	Euthyroid sick syndrome (*n* = 571)	Hypothyroidism (*n* = 150)	Subclinical hypothyroidism (*n* = 121)

Gender (*n*, %)^*∗*^					
Female	41 (6.3)	62 (9.6)	348 (53.8)	106 (16.4)	90 (13.9)
Male	14 (4.3)	12 (3.7)	223 (68.8)	44 (13.6)	31 (9.6)
Age (mean ± SD), years^*∗*^	44 ± 16.1	38 ± 14.6	49 ± 19.6	45 ± 15.3	48 ± 18.8
Age groups (*n*, %)					
18–30	11 (4.7)	28 (12)	140 (59.8)	30 (12.8)	25 (10.7)
31–50	29 (8.4)	32 (9.2)	166 (47.8)	73 (21)	47 (13.5)
51–70	11 (4.1)	11 (4.1)	176 (65.4)	36 (13.4)	35 (13)
71–90	4 (3.4)	3 (2.6)	86 (74.1)	11 (9.5)	12 (10.3)
≥91	0 (0.0)	0 (0.0)	3 (60)	0 (0.0)	2 (40)
Histopathological results (*n*, %)					
Thyroid cancer	1 (4.5)	0 (0.0)	11 (50)	5 (22.7)	5 (22.7)
Comorbidities (*n*, %)					
Malaria	6 (6.0)	1 (1.0)	63 (63)	15 (15)	15 (15)
HIV	3 (6.4)	0	30 (63.8)	4 (8.5)	10 (21.5)
Malaria + HIV	3 (7)	0	27 (62.8)	3 (7)	10 (23.5)
Diabetes mellitus	15 (11.5)	7 (5.4)	77 (59.2)	15 (11.5)	16 (12.3)

^*∗*^ *p* ≤ 0.001.

## Data Availability

The data used to support the findings of this study are available from the corresponding author upon request.
